# The dynamics of signal amplification by macromolecular assemblies for the control of chromosome segregation

**DOI:** 10.3389/fphys.2014.00368

**Published:** 2014-09-29

**Authors:** Semin Lee, Victor M. Bolanos-Garcia

**Affiliations:** ^1^Center for Biomedical Informatics, Harvard Medical School, Harvard UniversityBoston, MA, USA; ^2^Department of Biological and Medical Sciences, Faculty of Health and Life Sciences, Oxford Brookes UniversityOxford, UK

**Keywords:** spindle assembly checkpoint (SAC), genome instability, chromosome segregation, signal amplification, cell cycle regulation, kinetochore-microtubules network, cancer, protein-protein interactions

## Abstract

The control of chromosome segregation relies on the spindle assembly checkpoint (SAC), a complex regulatory system that ensures the high fidelity of chromosome segregation in higher organisms by delaying the onset of anaphase until each chromosome is properly bi-oriented on the mitotic spindle. Central to this process is the establishment of multiple yet specific protein-protein interactions in a narrow time-space window. Here we discuss the highly dynamic nature of multi-protein complexes that control chromosome segregation in which an intricate network of weak but cooperative interactions modulate signal amplification to ensure a proper SAC response. We also discuss the current structural understanding of the communication between the SAC and the kinetochore; how transient interactions can regulate the assembly and disassembly of the SAC as well as the challenges and opportunities for the definition and the manipulation of the flow of information in SAC signaling.

## The spindle assembly checkpoint (SAC)

The regulation of chromosome division in time and space requires amplification of specific signals across an intricate network of protein-protein interactions. Central to this process is the spindle assembly checkpoint (SAC), the essential and evolutionarily conserved self-regulatory system of the eukaryotic cell cycle that ensures accurate chromosome segregation by controlling cell cycle progression in response to microtubule-kinetochore attachment defects (Hardwick et al., 2000; Warren et al., 2002; Morrow et al., 2005; Yao and Dai, 2012; Foley and Kapoor, 2013; Jia et al., 2013). SAC function requires its communication with the kinetochore, the multiprotein network that assembles on mitotic or meiotic centromeres to link centromeric DNA with microtubules.

Three serine/threonine protein kinases, Bub1, BubR1, and Mps1 play essential roles in the mitotic checkpoint. Bub1 is required for the recruitment to the kinetochore, the site for attachment of chromosomes to microtubule polymers that pull sister chromatids apart during cell division, of several checkpoint components in cells that have the checkpoint unsatisfied. Bub1 is also important for the assembly of the inner centromere. BubR1 is required for the establishment of proper kinetochore-microtubule attachment and chromosome alignment and together with the proteins Bub3, Mad2, and Cdc20 forms part of the mitotic checkpoint complex (MCC) that inhibits the E3 ubiquitin ligase activity of the anaphase-promoting complex (also known as the cyclosome, APC/C) toward its substrates Securin and Cyclin B1 (Tang et al., 2004; Vanoosthuyse and Hardwick, 2005; Boyarchuk et al., 2007; Bolanos-Garcia and Blundell, 2011; Elowe, 2011; Chao et al., 2012). Mps1 is a dual-specificity kinase that localizes to kinetochores during mitosis and that through phosphorylation of kinetochore targets prevents aneuploidy by promoting both productive chromosome attachment and SAC function. Loss of Mps1 function in organisms from yeasts to humans overrides mitotic checkpoint signaling (Weiss and Winey, 1996; Abrieu et al., 2001; Maciejowski et al., 2010; Tipton et al., 2013). Mps1 has been identified in the signature of the top 25 genes overexpressed in tumors of different origins including bladder, anaplastic thyroid, breast, lung, esophagus, and prostate (Carter et al., 2006; Janssen et al., 2009). Recruitment of Bub1, BubR1, Mps1, Bub3, Ccd20, Mad1, and Mad2 to the kinetochore is essential for the full activity and optimal function of the mitotic checkpoint (revised in Musacchio, 2011; Hauf, 2013). APC/C inhibition is released after proper bipolar attachment and alignment of all chromosomes at the center of the cell, thus allowing chromosome separation and mitotic progression (revised in Jia et al., 2013).

Here we discuss the nature of protein-protein interactions underpinning mitotic checkpoint function, in which weak but cooperative association of individual protein components of the SAC to form larger, dynamic macromolecular assemblies has arisen as successful strategy to ensure the amplification of specific signals that control chromosome segregation in the crowded environment of the cell. We also discuss how emerging technologies and multidisciplinary strategies enable us to gain insights into SAC signaling with an unprecedented level of detail.

## Structural features of SAC protein components

**Figure 1:**
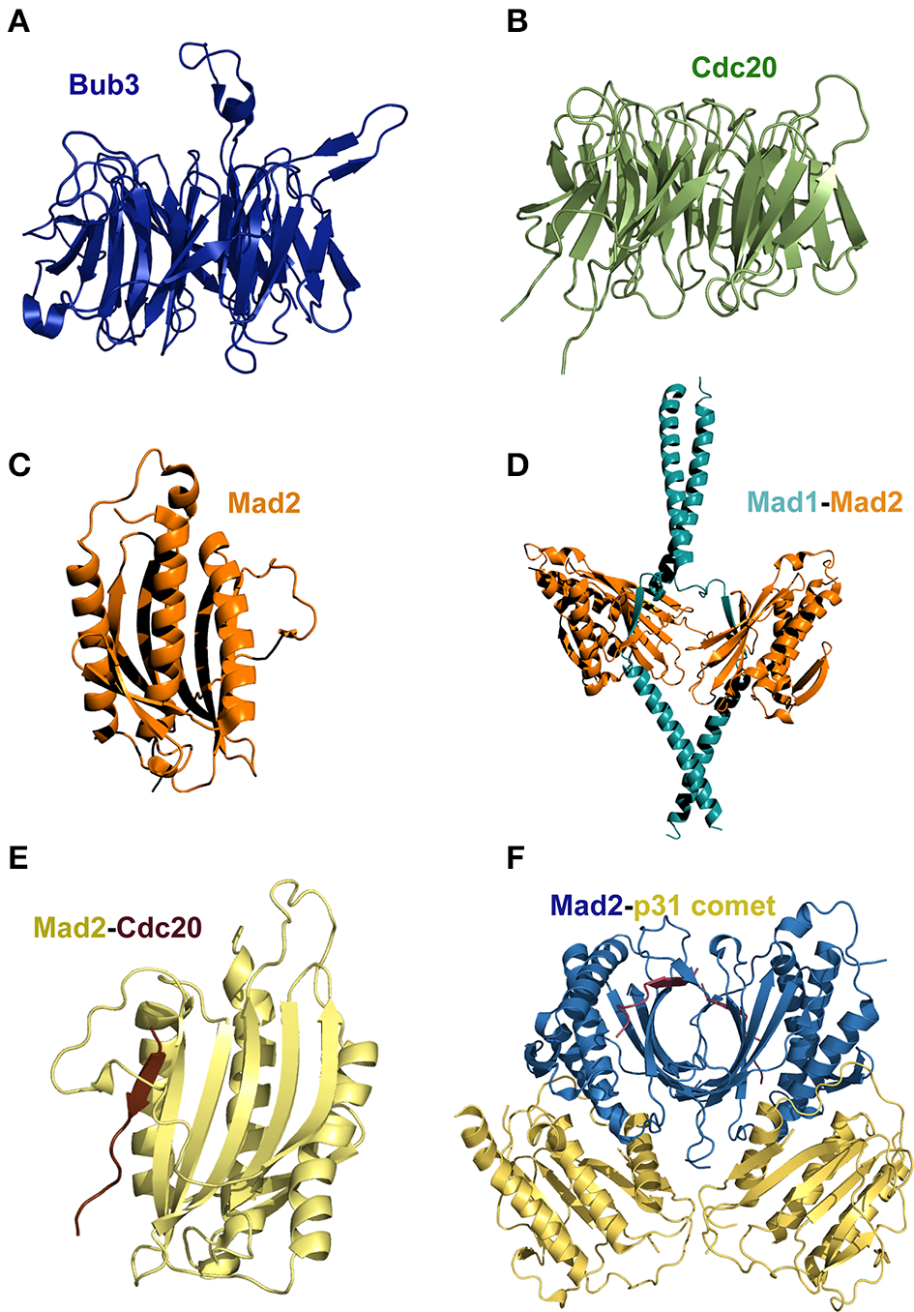
**(A)** Bub3 and **(B)** Cdc20 both adopt a seven-blades, WD 40 fold (pdb 1UAC and 4GGA, respectively). **(C)** The architecture of Mad2 defines a characteristic HORMA domain (pdb 1DUJ). **(D)** The structure of the Mad1-Mad2 complex shows that the two chains of Mad1 interact with Mad2 through the N-terminal coiled-coil region (pdb 1GO4). **(E)** Structure of a Cdc20 fragment bound to Mad2 (pdb 1KLQ). **(F)** Crystal structure of the Mad2/p31^comet^ complex (pdb 2QYF). A comparison of the latter two structures shows that p31^comet^ inhibits Mad2 activation through structural mimicry. Figures generated with PyMOL (DeLano, 2002).

Important clues into the inner working of the SAC have been derived from the structural understanding of central SAC components. For instance, the three protein kinases Bub1, BubR1 and Mps1, which share a common multidomain organization and play roles that are essential for the SAC, contain an N-terminal region that is organized as a tandem arrangement of the tetratricopeptide repeat (TPR) motif and a C-terminal kinase domain. In addition to Bub1, BubR1, and Mps1 kinases, the proteins Bub3, Mad1, Mad2, and Cdc20 also mediate key functions in SAC signaling. The crystal structure of Bub3 has shown that this protein is globular and contains a single domain (Larsen and Harrison, 2004; Wilson et al., 2005) that adopts the WD40-repeat fold. Despite its small size and presence of a single domain, Bub3 is known to physically interact with Bub1, BubR1, and Knl1. Another key molecule is Cdc20, a co-activator of the APC/C, the macromolecular assembly that is responsible for targeting proteins for ubiquitin-mediated degradation during mitosis (Nilsson et al., 2008; Izawa and Pines, 2012; Sedgwick et al., 2013), thus leading to the arrest of cells in mitosis (Musacchio and Salmon, 2007; Chao et al., 2012). Similar to Bub3, Cdc20 adopts the WD40-repeat fold (Figures 1A,B, respectively). However, in mammals Cdc20 also contains two independent degradation signals: the KEN box (Pfleger and Kirschner, 2000) and the CRY box (Reis et al., 2006). The former box is required for the APC/C dependent degradation of Cdc20 (Huang et al., 2001) whereas the CRY box (consensus amino acid sequence CRYxPS) functions as a second degradation signal in Cdc20 (Reis et al., 2006).

Mad1 is a predominantly coiled-coil protein that in humans encompasses 718 amino acid residues (Hardwick and Murray, 1995; Schuyler et al., 2012). Depletion of Mad1 in human cells results in genome instability and chromosome segregation defects (Luo et al., 2000; Maciejowski et al., 2010; Meyer et al., 2013) thus evidencing its essential role in the SAC (Luo et al., 2002). Mad2 adopts the HORMA (for Hop1, Rev7, and Mad2) domain (Luo et al., 2000) (Figure 1C). Mad2 binds to Mad1 to form a stable heterocomplex *in vitro* (Luo et al., 2002) that regulates the progression of mitosis by controlling the flow of Cdc20 into the SAC. In one hand, the Mad2-Mad1 heterocomplex binds to improperly attached kinetochores, inducing the hyper-phosphorylation and activation of Mad1 by Mps1 (Winey and Huneycutt, 2002; Hewitt et al., 2010). On the other hand, kinetochore bound Mad1-Mad2 catalyzes the assembly of a Mad2-Cdc20 complex (Figure 1D) (Sironi et al., 2001, 2002; Chung and Chen, 2002; De Antoni et al., 2005; Nezi et al., 2006; Mapelli et al., 2007; Yang et al., 2008; Kulukian et al., 2009; Lad et al., 2009; Fava et al., 2011) in a process that involves the conversion of Mad2 from an “open” into a “closed” Cdc20-bound conformation (Luo et al., 2000, 2004). How the above interactions lead to conformational transitions that contribute to regulate the segregation of chromosomes in space and time? This fascinating aspect of the SAC is addressed in the section below.

## Dynamics of macromolecular assembly/disassembly

Earlier clues of the dynamic nature of the network of interactions underpinning SAC signaling were provided by the crystal and NMR structures of members of the Mad protein family. For instance, Sironi and collaborators reported the structure of a Mad1-Mad2 complex that revealed a loop around the Cdc20 binding site of Mad2 (Figure 1D) and suggested a “safety-belt” mechanism underlying the regulation of the interactions between Mad2-Mad1 and Mad2-Cdc20 (Figure 1E) (Sironi et al., 2002). Further structural details of Mad2 transitions between an “open” and a “closed” conformational state have been established by X-ray protein crystallography and NMR (Luo and Yu, 2008; Kim et al., 2010; Li et al., 2014). The 3D structures show that the transition of Mad2 from the “open” to the “closed” conformation involves a large conformational rearrangement of the polypeptide chain. This dramatic conformational switch is regarded as the rate-limiting step in cells mounting a SAC response (De Antoni et al., 2005; Vink et al., 2006; Hewitt et al., 2010; Maldonado and Kapoor, 2011; Lau and Murray, 2012).

**Figure 2:**
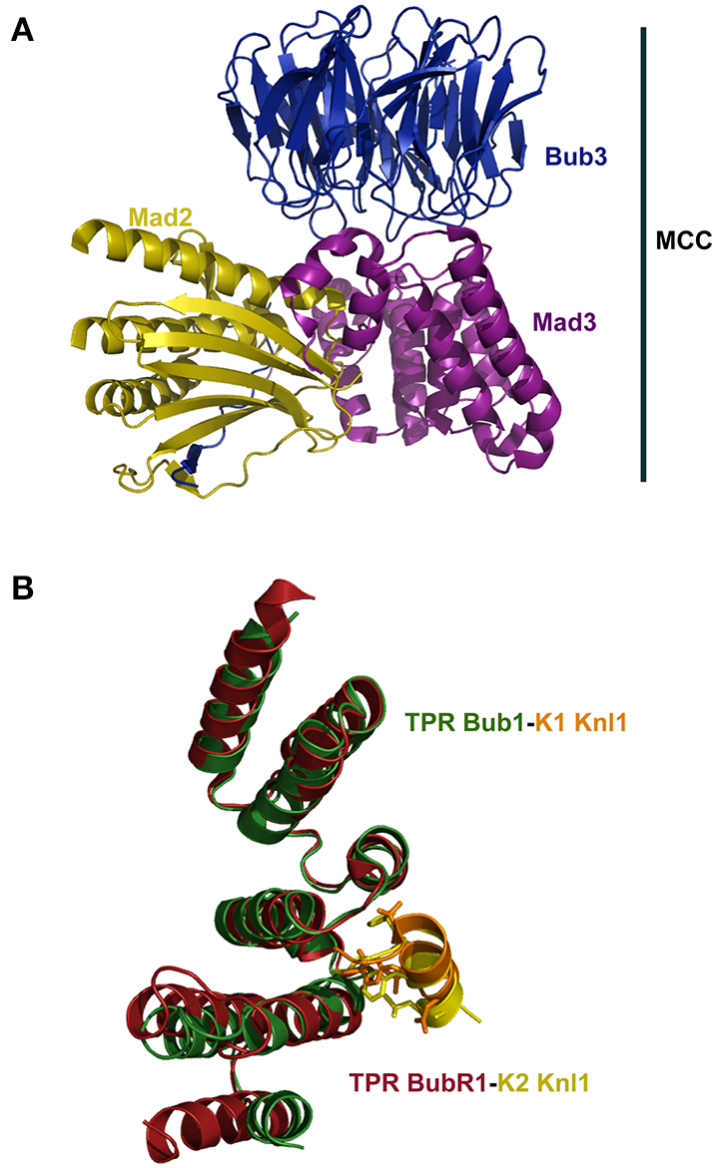
**(A)** Crystal structure of the Mitotic Checkpoint Complex (MCC) from *Schizosaccharomyces pombe* (pdb 4AEZ). **(B)** The N-terminal regions of Bub1 and BubR1 are organized as a triple tandem of the TPR motif. Superposition of the structures of TPR Bub1 and TPR BubR1 in complex with the KI motifs of Knl1 (pdb 4AIG and 3SI5, respectively) revealed a similar mode of binding underlying disorder-to-order transitions.

SAC signaling is antagonized by the protein p31^comet^ (Habu et al., 2002; Xia et al., 2004). The crystal structure of the closed conformation of Mad2 in complex with p31^comet^ showed that the latter protein interacts extensively with the Mad2 dimerization interface in such a way it inhibits the conformational transition to the Mad2 open state (Yang et al., 2007). The binding of Mad3 (the yeast BubR1 homolog that lacks the catalytic kinase domain) and p31^comet^ to the same Mad2 interface implies a competition between p31^comet^ and Mad3 to bind Mad2 (Figure 1E). The structure of the p31^comet^-Mad2 complex (Figure 1F) thus provides structural insights into the regulation of MCC assembly and disassembly. Furthermore, the crystal structure of the mitotic checkpoint complex (MCC, Figure 2A) from fission yeasts revealed the mode in which Mad2 and Mad3 cooperate to inhibit Cdc20 (Chao et al., 2012). The MCC structure shows that Mad2 and Mad3 complex formation facilitates the presentation of the KEN box motif of Mad3 to the KEN-box motif of Cdc20 (Figure 2A). Interestingly, an unexpected D-box mimic located at the C-terminal end of Mad3 revealed the D-box-binding site on Cdc20, which provided the first structural insight into the mechanism of degron recognition by co-activators (an aspect of SAC signaling that has been nicely revised by Zhang et al., 2014). The structure of the MCC shows that APC/C ubiquitin ligase activity is modulated by steric hindrance that impedes substrate recognition and also through conformational changes that disrupt the architecture of the substrate-binding site. Such mode of regulation closely resembles the molecular mechanisms underlying the control of protein kinases (Chao et al., 2012). This mode of regulation is in sharp contrast with the mechanism of regulation of other signaling systems such as the SCF (SKP1-Cullin1-F-box-Rbx1) complex. In the latter case, the E3 ubiquitin ligase activity of SCF is regulated at the level of substrate recognition in a process that involves phosphorylation of a degradation signal (degron) consensus motif, IL-I/L/PpT-P, that is present on substrates targeted for proteasomal degradation (Welcker and Clurman, 2008; Zhou et al., 2013).

## SAC communication with the KMN network

SAC function requires its communication with the kinetochore, the multiprotein complex that is assembled on mitotic or meiotic centromeres to connect centromeric DNA with microtubules (Funabiki and Wynne, 2013; Westhorpe and Straight, 2013). Although the assembly of the kinetochore is a crucial event in cell division, the precise sequence of events underlying the process remains obscure. As discussed below, recent structural insights show that the establishment of kinetochore complexes often involves dramatic conformational changes, including disorder-to-order transitions. Although the amino acid sequence in most kinetochore proteins is clearly divergent, the overall architecture of the kinetochore remains highly conserved across species (Przewloka and Glover, 2009; Tanaka, 2013; Westhorpe and Straight, 2013). The structural core of the kinetochore is the KMN network, which constitutes a docking platform for the kinetochore recruitment of SAC components. The KMN network is composed by the single protein **K**nl1 (a protein also known as CASC5, Blinkin, and AF15Q14 in humans; Spc105 in budding yeast and flies and Spc7 in fission yeast) (Kiyomitsu et al., 2007, 2011; Bolanos-Garcia et al., 2009) and the protein complexes **M**is12/Mtw1/MIND and **N**dc80/HEC1. The latter two assemblies are commonly referred to as the Mis12 and Ndc80 complexes.

Knl1 is a large, predominantly disordered protein of the KMN network that acts as molecular platform for the recruitment of several proteins to the kinetochore (Kiyomitsu et al., 2007; Przewloka and Glover, 2009; Santaguida and Musacchio, 2009; Ghongane et al., 2014). In mammals, depletion and/or suppression of the expression of Knl1 lead to extensive chromosome missegregation with phenotypes that closely resemble those caused by depletion of Bub1 and BubR1 kinases (Cheeseman et al., 2006, 2008; Kiyomitsu et al., 2007). Knl1 plays a central role in the dynamics of the assembly/disassembly of the KMN network and directly interacts with a range of proteins that are essential for proper chromosome segregation, including Protein phosphatase 1 (Pp1), Bub1, BubR1, Bub3, Zwint, and Nsl1, a component of the Mis12 complex. Such complex choreography of interactions confers exquisite regulation to the SAC. For example, the very N-terminal end region of Knl1 recruits Pp1 to the kinetochore; another N-terminal segment binds to the TPR motifs of Bub1 and BubR1 (Figure 2B) (Bolanos-Garcia et al., 2011; Rosenberg et al., 2011; Krenn et al., 2012; London et al., 2012; Shepperd et al., 2012; Funabiki and Wynne, 2013) whereas C-terminal Knl1 binds directly to Nsl1 (Primorac et al., 2013; Petrovic et al., 2014) and possibly also to Dsn1, another protein component of the Mis12 complex (Cheeseman et al., 2006; Kiyomitsu et al., 2007). The majority of Knl1 homologs contain an arrangement of motif repeat units, the MELT motif, that thus far seem to be a unique feature of this kinetochore docking platform. The specific amino acid sequence and the number of MELT repeat units is widely variable across species thus suggesting that the differences contribute to the specie-specific recognition of different Knl1 partners. A comparison of the buried area upon complex formation between SAC proteins and between SAC-KMN components show a relatively small buried area, ranging from approx. 500 to 1700 Å^2^ (Table 1), values that are similar to those calculated for non-obligate complexes (Jones and Thornton, 1996). It has been shown that the binding of Spc105, the fly homolog of Knl1, to Nnf1a, Nnf1b, Nsl1, and Mis12 is interdependent as the proteins need of each other for their recruitment to the kinetochore (Venkei et al., 2012). Although such feature suggests a cooperative mode of interaction between these proteins, it would be important to define more precisely the dynamics and stoichiometry of the interactions and to establish if a similar interdependence for their recruitment to the kinetochore occurs in other species.

**Table 1:** **Analysis of interface area in Å^2^, calculated as difference in total accessible surface areas of isolated and interfacing structures divided by two, according to ePISA (Protein Interfaces, Surfaces and Assemblies) tool (EMBL-EBI, UK)**.

**Protein complex**	**Protein-protein interface area (Å^2^)**	**PDB code**	**Reference**
Bub1-GLEBS motif of Bub3	1664	2I3S	Larsen and Harrison, 2007
Mad3-GLEBS motif of Bub3	1681	2I3T	Larsen and Harrison, 2007
Bub1-Bub3 in ternary complex	1655	4BL0	Primorac et al., 2013
Bub3-MELT motif of Knl1 in ternary complex	654	4BL0	Primorac et al., 2013
Bub1-KI-1motif of Knl1	527	4A1G	Krenn et al., 2012
BubR1-KI-2 motif of Knl1	464	3SI5	Bolanos-Garcia et al., 2011
Nsl1-RWD domain of Knl1	565	4NF9	Petrovic et al., 2014

## The importance of low structural complexity in the SAC

The organization of a polypeptide chain in regions that exhibit low structural complexity is a recurrent feature of protein molecules (Dunker et al., 1998; Dyson and Wright, 2002, 2005; Gsponer and Babu, 2009; Babu et al., 2012). A bioinformatics study helps to illustrate this as it shows that 35–51% of eukaryotic proteins have at least one disordered region that span fifty or more amino acid residues (Dunker et al., 2002). The Protein Disorder Database DisProt (http://www.disprot.org; Sickmeier et al., 2007) provides additional support this view. Up to date (last release date 05/24/2013) the database has annotated 1539 disorder regions in a total of 694 proteins. Importantly, diverse bioinformatics studies have demonstrated that large polypeptide segments of low structural complexity are abundant in proteins that act as docking platforms for the binding of multiple partners (Dunker et al., 2005; Dosztanyi et al., 2006; Haynes et al., 2006; Kim et al., 2006a). The highly flexible surfaces of regions of low structural complexity can be critical for the formation of productive macromolecular complexes (Dyson and Wright, 2005; Kim et al., 2006a; Schlessinger et al., 2007; Dunker et al., 2008). Indeed, it has been established that disordered binding regions play a critical role in diverse biological processes (Dyson and Wright, 2002) and that the association of individual proteins to form macromolecular assemblies can have a profound effect on the stability; transport properties; subcellular localization of the complexes and affect further interactions with additional molecules and/or assemblies (Sasahara et al., 2003; Banks and Fradin, 2005; McGuffee and Elcock, 2010; Wang et al., 2010, 2012; Cino et al., 2012; Miermont et al., 2013). In principle, large polypeptide segments of low structural complexity in hub proteins including Knl1 and other components of the KMN network can allow different conformers of the same polypeptide chain to bind with different affinity to interacting partners. An interesting suggestion is that interactions mediated by certain protein families involve the binding to specific linear motifs that capture key residues responsible for the interactions. Such linear motifs have been categorized and used to complement the prediction of binding sites in regions of low structural complexity with specific motif searches (Puntervoll et al., 2003). One interesting property of regions of low structural complexity is the transition from a disorder to a more ordered state upon ligand binding (a feature also known as coupled folding and binding). Examples of this class of transitions in SAC signaling occur upon binding of N-terminal Bub1 and BubR1 to KI motifs of Knl1; the interaction of Mad2 with Mad1 and Cdc20 and Bub3 binding to the MELT motifs of Knl1, to name a few. A more detailed discussion of the importance of this mode of binding in the SAC is show below, in the section entitled disorder-to-order transitions. Intrinsic disorder proteins seem prone to initiate promiscuous molecular interactions when over expressed and that for this reason they can contribute to toxicity/pathology (Vavouri et al., 2009). Interestingly, the structural properties of intrinsic disorder proteins seem to correlate strongly with the observed dosage sensitive (i.e., give place to a pathological condition when the expression is increased) of oncogenes, suggesting that mass action driven molecular interactions may be an important cause of cancer (Vavouri et al., 2009). Because dosage-sensitive genes seem to be slightly enriched in those mediating cell cycle regulation (Sopko et al., 2006), it would be important to define the dosage sensitive of genes associated with SAC signaling and its contribution (if any) to the onset of chromosome segregation defects and/or aneuploidy.

## DNA compaction and crowding effects

As discussed by Burgess and collaborators in their excellent mini-review, the repair of DNA damage during mitosis is generally difficult due to the suppression of gene transcription and translation caused by the level of DNA compaction (Burgess et al., 2014). For example, little is known about the effect of centromeric DNA compaction on the assembly of the kinetochore. What is known is that DNA binding to the kinetochore does not depend on a specific DNA sequence (with a few exceptions) and that the deposition of Cenp-A-containing nucleosomes at the centromeric chromatin is likely to rely on epigenetic mechanisms. However, definition in greater detail of the extent in which centromere identity is specified by epigenetic mechanisms remains a central question in the study of chromosome inheritance and genome stability.

**Figure 3:**
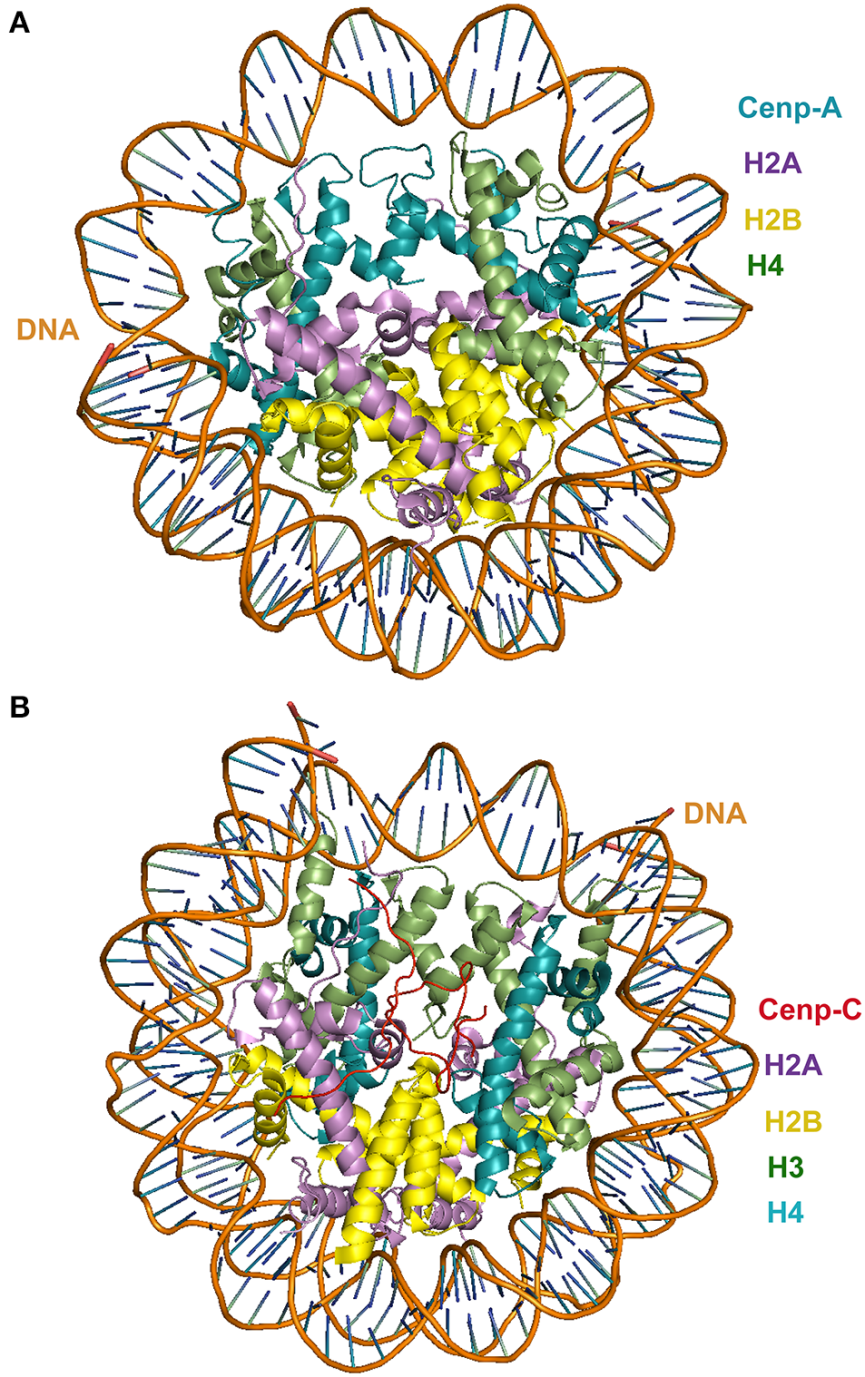
**(A)** Crystal structure of Cenp-A in complex with centromeric nucleosome; **(B)** crystal structure of Cenp-C in complex with centromeric nucleosome. In both cases the view is in the axis of the DNA supercoil.

It has been established that a constitutive complex, the centromere-associated network (CCAN), is assembled onto centromeric Cenp-A chromatin. The CCAN consists of 16 proteins: Cenp-C, Cenp-H/Cenp-I/Cenp-K, Cenp-L/Cenp-M/Cenp-N, Cenp-O/Cenp-P/Cenp-Q/Cenp-R/Cenp-U, Cenp-T/Cenp-W, and Cenp-S/Cenp-X (revised by Perpelescu and Fukagawa, 2011). CCAN recruits the outer kinetochore components of the KMN network Knl1, the Mis12 complex, and the Ndc80 complex thus linking structural and regulatory kinetochore proteins which spindle microtubules. Cenp-A, a conserved centromere-specific variant of the protein histone H3 (Palmer et al., 1991; Stoler et al., 1995; Perpelescu and Fukagawa, 2011), plays a role in the propagation of centromere identity and the formation of the kinetochore (Barnhart et al., 2011; Mendiburo et al., 2011; Fachinetti et al., 2013). This manner, the centromere-kinetochore assembly guides the movement of chromosomes and the progression of the cell cycle throughout mitosis (Wan et al., 2009). Cenp-C and Cenp-T, two components of the CCAN, are required for spindle attachment. Structural insights of the human centromeric nucleosome containing Cenp-A in complex with its cognate α-satellite DNA derivative revealed that in the human Cenp-A nucleosome, the DNA wraps around a histone octamer comprising two molecules of histones H2A, H2B, H4, and Cenp-A (Tachiwana et al., 2011). The crystal structure of the Cenp-A nucleosome (pdb ID 3AN2) supports the octasome model (Figure 3A). However, the existence of a Cenp-A nucleosome complex comprising one of each core histone (a complex referred to as the hemisome) has been suggested (Tachiwana et al., 2011). The two different complexes may not be mutually exclusive as there is a possibility both the octasome and the hemisome can be assembled *in vivo*. This is an aspect that should be clarified if we are to understand the precise role of Cenp-A in the control of chromatin assembly and its influence in the formation of the kinetochore.

Interestingly, among all the protein that are known to associate constitutively with human Cenp-A chromatin, only Cenp-C has been identified in all model organisms (Stoler et al., 1995). Human Cenp-C consists of four functional regions (Figure 3B). The N-terminal region binds to the Mis12 complex (Barnhart et al., 2011). The central region of Cenp-C is required for the targeting of the protein to the centromere (Fachinetti et al., 2013) in a process that involves the recognition of the carboxyl tail of Cenp-A in the centromeric nucleosome (Mendiburo et al., 2011). The C-terminal region of Cenp-C is responsible for homo dimerization of the protein (Hori et al., 2013). The specific recognition of the histone variant Cenp-A in the centromeric nucleosome by Cenp-C is critical for the assembly of the kinetochore. The crystal structure of Cenp-C in complex with the nucleosome core particle (pdb 4INM) has revealed the determinants of the recognition mechanism. The structure shows that Cenp-C binds a hydrophobic region in the Cenp-A tail and docks onto the acidic patch of histone H2A/H2B. The Cenp-C-nucleosome core particle complex thus revealed a conserved mechanism for recruitment of proteins to centromeres. It also provides insights into the molecular mechanism of histone recognition in which a disordered peptide binds the histone tail. Such mode of nucleosome docking is facilitated by extensive hydrophobic interactions, a structural feature also observed in diverse SAC and kinetochore assemblies that involve disorder-to-order transitions, an aspect that is discussed in more detail in the next section.

Cenp-E is a member of the Cenp protein family (Perpelescu and Fukagawa, 2011; Przewloka et al., 2011) that, similar to Cenp-C and Knl1, contains large segments of low structural complexity throughout the polypeptide chain. Cenp-E functions as a plus-end directed molecular kinesin-like motor protein that is localized specifically to kinetochores during mitosis and that is required for efficient capture and attachment of kinetochores to the spindle microtubules (McEwen et al., 2001; Putkey et al., 2002; Kapoor et al., 2006). In human cells, Cenp-E depletion by RNA interference (Tanudji et al., 2004) or antisense oligonucleotides (Yao et al., 2000) and inhibition of its recruitment to kinetochores by antibody microinjection (Schaar et al., 1997; McEwen et al., 2001) result in chromosome congression aberrations. The intrinsic structural flexibility of Knl1, Cenp-C, Cenp-E and other kinetochore proteins should facilitate the establishment of productive and specific interactions with diverse interacting partners (Mao et al., 2003). In a broader sense, the recurrence of regions of low structural complexity in SAC and KMN protein components is likely to play a critical roles in the control of chromosome segregation none less because greater selectivity can be achieved through interactions that involve multiple components.

Macromolecular crowding refers to the confinement in the cellular space of macromolecules at high concentration (Elcock, 2010; Hancock, 2012). Studies carried out in mice showed that abnormal higher Mad2 levels lead to aberrant SAC function and induced tumorigenesis (Sotillo et al., 2007, 2010; Schvartzman et al., 2011). It would be important to establish to what extent Mad2 crowding contributes to the above-mentioned abnormal processes.

Some proteins can process distinct molecular signals under the crowding conditions of the cell. An example of this phenomenon is transport kinesins (such as kinesin-1), which seems to have evolved molecular properties that prevent it from forming traffic jams in the crowded conditions of the cells (Leduc et al., 2012) and the kinesin motor protein kinase ERK, which can be phosphorylated in a processive manner in HeLa cells (Aoki et al., 2011). Interestingly, under conditions that recreate physiological molecular crowding, the mode of ERK phosphorylation shifts from distributive to processive (Aoki et al., 2011), in which case ERK does not form a stable substrate-enzyme complex, a behavior that is the opposite to that observed in the processive phosphorylation model. It would be important to establish if phosphorylation shifts from distributive to processive or vice versa occurs in components of the KMN network such as Knl1, Cenp-C, and Cenp-E.

## Disorder-to-order transitions

**Figure 4:**
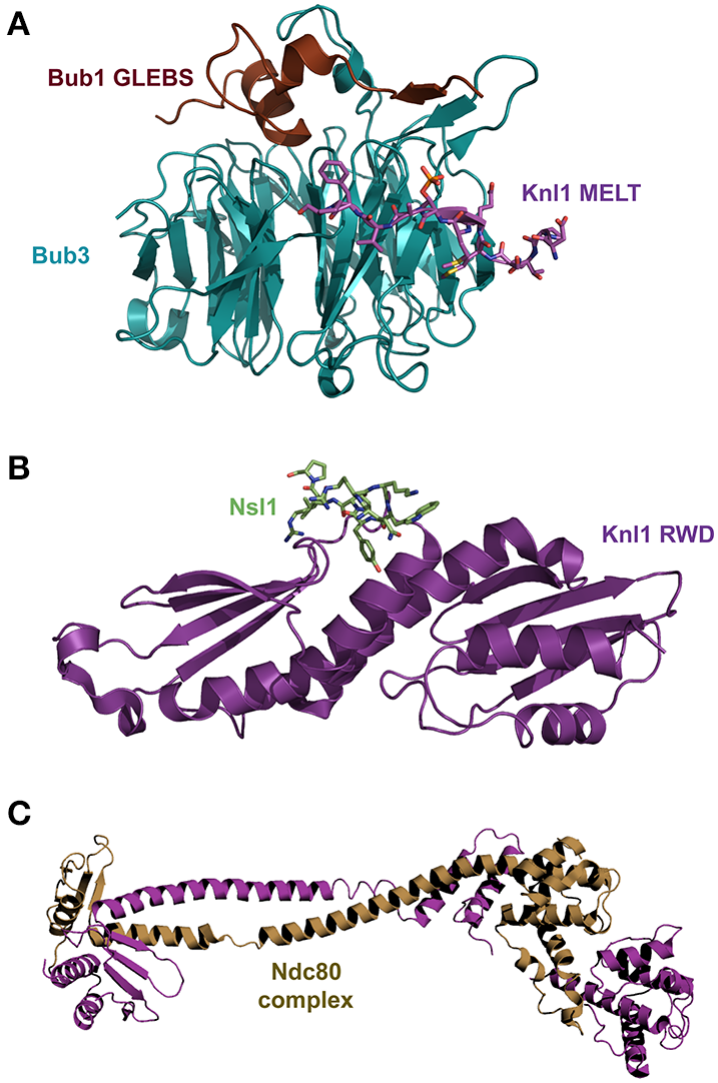
**(A)** Superposition of the crystal structure of the Bub3-Bub1 GLEBS motif-Knl1 MELT motif ternary complex (pdb 4BL0). **(B)** Knl1 RWD domain in complex with Nsl1 (pdb 4NF9). **(C)** Crystal structure of a chimeric (bonsai) Ndc80 complex (pdb 2VE7).

Comparison of the structures of diverse SAC and kinetochore complexes reveals a recurrent mode of binding that is characterized by disorder-to-order transitions. Examples of this class of transitions occur in the interaction of Mad2 with Mad1 and Cdc20; the binding of TPR domains of Bub1 and BubR1 to KI motifs of Knl1 (Figure 2B); the binding of Bub3 to the MELT motifs of Knl1 (Figure 4A) and the binding of the RWD domain of Knl1 to a synthetic peptide that mimics Nsl1 (Figure 4B) (Bolanos-Garcia et al., 2011; Kiyomitsu et al., 2011; Krenn et al., 2012; Primorac et al., 2013; Petrovic et al., 2014; revised in Ghongane et al., 2014). In all these complexes the binding of an otherwise predominantly disordered protein fragment to the globular partner involves dramatic conformational transitions that lead to the formation of an α-helix upon complex formation. The predominance of cooperative, stabilizing hydrophobic interactions is another structural feature that emerges from the analysis of the aforementioned complexes, where only little conformational changes are observed in the BUBs after complex formation.

The interaction of SAC kinases Bub1 and BubR1 with the protein Knl1 physically links SAC signaling with the kinetochore (Kiyomitsu et al., 2007, 2011; Bolanos-Garcia et al., 2011). The crystal structure of N-terminal Knl1 with TPR BubR1 defines an extensive hydrophobic interface in which a mechanistic zipper mode of binding has been suggested. In this model, several Knl1 residues (I213, F215, F218, and I219) sequentially dock into BubR1 pockets, thus ensuring high specificity and sensitive regulation. Furthermore, comparison of the crystal structure of the TPR BubR1-Knl1 binary complex with free Knl1 peptides titrations using 2,2,2-trifluoroethanol and monitored by far-UV circular dichroism revealed a disorder-to-order transition of N-terminal Knl1 upon binding BubR1. This is possible because a hydrophobic environment can be mimic experimentally with 2,2,2-trifluoroethanol, a solvent of low dielectric constant, ε, (ε = 8.55). The observed disorder-to-order transition of N-terminal Knl1 when binding to BubR1 can be expected for the interaction of Knl1 with Bub1, given the similar mode of binding (Figure 2B). Importantly, the local conformational changes trigger by disorder-to-order transitions upon BUBs binding should influence the interaction of Knl1 with other interacting partners such as specific kinases and/or phosphatases thus contributing to the regulation of the SAC (Liu et al., 2010; Rosenberg et al., 2011).

Bub1 and BubR1 (Mad3 in yeast) have a conserved stretch of about 40 amino acid residues downstream the N-terminal TPR domain that is predicted to be of low structural complexity and that harbor a Bub3 binding region commonly referred to as the GLE2p-binding sequence (GLEBS) motif. The crystal structures of two independent complexes formed between the GLEBS motifs of Mad3 and yeast Bub1 with Bub3 show the establishment of an extensive interface along the top surface of Bub3 upon complex formation (Larsen and Harrison, 2007) (Figure 4A). Such mode of binding implies a large conformational shift of the GLEBS motifs from a disorder to an ordered state. In a similar fashion, the crystal structure of a Mad1 fragment (residues 485–584) in complex with Mad2 revealed that the Mad1 fragment adopts a predominantly α-helix conformation upon complex formation (Luo et al., 2000) (Figure 1D). Furthermore, binding studies *in vitro* suggest an important conformational transition in which Mad1 primes the Mad2 binding site for the interaction with Cdc20 (Luo et al., 2002). *In vivo*, such concerted conformational rearrangements should ensure the tight regulation of the APC/C's ubiquitin-ligase activity (Tang et al., 2001; Jia et al., 2013).

## SAC-KMN signal amplification by the means of weak, cooperative interactions

Because multiprotein complexes that form cooperatively would less likely to be formed fortuitously (Blundell et al., 2002; Bolanos-Garcia et al., 2012), the cooperative association of higher order SAC signaling complexes resulting from binary interactions that are both specific and of low-affinity should favor the amplification of specific signals to mount an effective SAC response. The cooperative assembly of the KMN subcomplexes Mis12 and Ndc80 illustrates how the establishment of higher order signaling complexes can regulate the SAC. The Ndc80 subcomplex is composed of four subunits: Ndc80 (the subunit that gives its name to the entire subcomplex), Nuf2, Spc24, and Spc25 (Ciferri et al., 2005, 2008; Wei et al., 2005, 2007; Wan et al., 2009). The Ndc80 subcomplex adopts a dumbbell shape molecule with Spc24-Spc25 and Nuf2-Ndc80 located in opposite ends of the molecule (Figure 4C) (Ciferri et al., 2005; Wei et al., 2005). The association of Nuf2-Ndc80 is required for the binding of the Ndc80 complex to microtubules while the formation of the Spc24-Spc25 heterodimer is required for binding Knl1 and the Mis12 complex (Cheeseman et al., 2006; Kiyomitsu et al., 2007; Wei et al., 2007; Ciferri et al., 2008; Joglekar and DeLuca, 2009; Wan et al., 2009).

The exquisite regulation of the SAC is a fine example of how the remodeling of macromolecular assemblies in time and space has evolved as a successful strategy that increases selectivity of signals with a minimal margin for errors. At the same time, the highly versatile and dynamic remodeling of macromolecular assemblies constitutes a great challenge for their functional, biochemical and structural characterisation in space and time. Furthermore, a wide range of post-translational modifications such as acetylation, phosphorylation, ubiquitylation and sumoylation can have a significant impact on protein stability, turnover, reversibility, sub-cellular localisation and the hierarchical order of assembly/disassembly of protein complexes thus constituting and additional layer of control of cell signaling (Pawson and Nash, 2003; Kim et al., 2006b; Seet et al., 2006; Simorellis and Flynn, 2006; Mao et al., 2011; Wan et al., 2012; Jia et al., 2013).

## New approaches to the study of SAC macromolecular assemblies

Our discussion of the interactions underpinning SAC signaling is typical of many cell regulation systems, where a large number of macromolecules tend to associate, thus requiring the ability to describe the dynamics of transient complex formation and dissociation in both space and time. One strategy to achieve this is to combine a range of biophysical and biochemical methods with spatial techniques for structural biology. For example, time-resolved Raman scattering and X-ray scattering can be very powerful to study the dynamics of macromolecular interactions when they are combined with X-ray protein crystallography, Nuclear Magnetic Resonance (NMR), Small Angle X-ray Scattering (SAXS), and Electron Microscopy (EM). A useful approach to the study of the dynamic of macromolecular complexes underpinning the SAC-kinetochore-microtubule interactome is the stabilization and fixation of the complexes which can be achieved by incorporation of phospho-mimicking mutations; truncation or extension of the polypeptide chain; the addition of post-translational modifications, such as phosphorylation, acetylation, methylation and the use of crosslinking agents, to name just a few. The stabilization and fixation of complexes can be combined with Förster resonance energy transfer (FRET) to define temporal aspects of the interactions but also local conformational changes associated with SAC signaling. Importantly, exiting new experimental strategies for the study of dynamic systems are currently in fast development. For example, free-electron lasers (FEL) a technique that relies on the generation of X-ray pulses of very high intensity and short duration, has facilitated the structural determination of macromolecular complexes even from very small crystals of relatively low quality. The ultrashort X-ray flashes ensure that the molecules hardly change during the exposure and enable the study of functional processes through the monitoring of the motion of molecules from instant to instant. This is particularly attractive to the study of the interactions underpinning the SAC where is important to closely follow the dynamics of the association and dissociation of macromolecular assemblies. Current free-electron lasers facilities are the European X-ray free-electron laser, the Linac Coherent Light Source (LCLS) at the SLAC National Accelerator Laboratory, the Free electron LASer in Hamburg (FLASH), the SPring-8 Compact SASE Source (SCSS), and the PSI SwissFEL. Another exciting new development is transmission electron microscopy (TEM). A TEM variant that uses cryo-technology (Cryo-TEM) permits a full range of semi-automated applications, including 2D electron crystallography, single particle analysis, cryo electron microscopy, and dual-axis cellular tomography of frozen hydrated cell organelles and cells. Cryo-TEM, when combined with protein X-ray crystallography, NMR and molecular modeling studies, facilitates the generation of complete atomic models. Additional advantages of cryo-TEM are: (1) is its suitable to study complexes that are 250 kDa or larger; (2) it can be applied to the study of heterogeneous samples and (3) it can provide structural details of dynamic complexes, such as those defining the architectures of the kinetochore and the nucleosome, that are difficult to examine with other structural biology techniques.

A major challenge will be to extend the analysis of structure and dynamics of isolated SAC and kinetochore assemblies to the understanding of the organization of signal generation and amplification in the cell in space and time. Because large multiprotein complexes play critical roles in cell regulation, interfering with the dynamics of their assembly and/or dissociation rises as an attractive strategy for the treatment of diseases.

## Closing remarks

The function and regulation of the SAC depends upon a hierarchical organization of macromolecular assemblies in time and space to ensure the accurate and timely transmission of the genetic material to descendants. A common theme emerging from the structural analysis of SAC complexes is the adoption of a regular structure by one of the interaction partners upon complex formation.

SAC components that are intrinsically disordered in the unbound form often associate to binding partners with low affinity but high specificity thus mounting an effective SAC response. Interaction with one or more ligands through multiple linear motifs is an effective strategy to control the flow of information and to modulate the signal. Therefore, the greater selectivity that communication of the SAC with the KMN network demands is gained by the involvement of multiple components that assemble in a cooperative fashion. Undoubtedly, the structural characterisation of larger SAC protein assemblies will reveal novel molecular details of how signal amplification is achieved to control chromosome segregation in higher organisms.

Therefore, the timely assembly of protein subcomplexes in which at least one of the components shows low structural complexity appears a reiterate structural feature in SAC signaling.

Importantly, regulation of this critical cellular process relies on the establishment of transient interactions in space and time. This manner, multi-protein assemblies can associate cooperatively to confer high selectivity and sensitivity to the interactions.

Undoubtedly, the detailed knowledge of the architecture of large SAC and kinetochore complexes will provide the structural basis for the rational targeting of specific protein-protein interfaces with drugs, being these small size molecules, peptides, nucleic acids or carbohydrates.

### Conflict of interest statement

The authors declare that the research was conducted in the absence of any commercial or financial relationships that could be construed as a potential conflict of interest.
